# Recurrent facial keratoacanthoma in a patient with diabetes: a case report

**DOI:** 10.1186/1756-0500-7-257

**Published:** 2014-04-23

**Authors:** Esam Omar

**Affiliations:** 1 Oral & Maxillofacial Surgery, College of Dentistry, Taibah University, Almadinah, Saudi Arabia

**Keywords:** Report, Recurrent keratoacanthoma

## Abstract

**Background:**

Keratoacanthoma is a relatively common low-grade malignancy that originates in the pilosebaceous glands. Pathologically, it closely resembles squamous cell carcinoma. Keratoacanthoma is believed to have a good prognosis; however, it has been reclassified as squamous cell carcinoma, keratoacanthoma type, to reflect the difficulty in histologic differentiation of this lesion as well as its uncommon but potentially aggressive nature. Keratoacanthoma infrequently presents as multiple tumors and may enlarge (5–15 cm), become locally aggressive, and rarely metastasize.

**Case presentation:**

A 66-year-old Arab male patient with diabetes was referred to the Maxillofacial Surgery Department with a chief complaint of a dome-shaped nodule with a smooth, shiny surface and central crateriform ulceration with a keratin plug in the form of a horn-like projection. Skin papules were present in the right lateral canthal area and extended to the lateral border of the lower eyelid, measuring 1.3 cm. On palpation, the lesion was firm, movable, and tender. The patient had a history of a similar lesion in another area of his face that had spontaneously regressed.

**Conclusion:**

Histological differentiation between keratoacanthoma and well-differentiated squamous cell carcinoma is difficult, particularly when a secondary infection is present. Careful establishment of the correlation among the history, clinical findings, and histopathology is highly indicated to avoid unnecessary surgical intervention.

## Background

Keratoacanthoma (KA) is a relatively common low-grade malignancy that originates in the pilosebaceous glands. Pathologically, it closely resembles squamous cell carcinoma (SCC). KA is characterized by rapid growth over a few weeks to months, followed by spontaneous resolution over 4 to 6 months in most cases. It is noninvasive and does not metastasize. Both chemical carcinogens and sunlight have been implicated as pathologic factors involved in the growth of the tumor. Genetic factors, human papilloma virus, trauma, and an immunocompromised status have also been implicated as etiologic factors [1-6]. Similar epidemiological features between KA and conventional SCC suggest a possible common pathogenesis, such as actinic damage [7]. KA and SCC have comparable incidences [7]. After the age of 64 years, the incidences of both KA and SCC increase significantly. The average patient age at the time of lesion development is 67 years for KA and 66 years for SCC. The male:female ratio for both conditions is similar at 2:1 [7]. Chuang et al.*,*[8] and Reizner et al.*,*[9] reported that the incidence of KA is equal to that of SCC and challenged the commonly reported KA:SCC incidence ratio of 1:3. The peak incidence occurs in the seventh decade or beyond. In other studies, the ratio of KA to SCC has ranged from 1.0:0.6 to 1:5 in different geographic locations [8,9]. KA is uncommon in darker-skinned patients. Approximately one-fifth to one-sixteenth of cases affect American whites [8,9]. Differentiation of KA from well-differentiated SCC may be problematic. The malignancy, however, lacks the characteristic acute angle between the normal and lesional epithelial margins of the KA, and both benign entities demonstrate a “pushing” extension into the underlying stroma with none of the characteristic invading islands and individual dysplastic cells of the carcinoma.

## Case presentation

A 66-year-old Arab male patient with diabetes was referred to the Maxillofacial Surgery Department with a chief complaint of a dome-shaped nodule with a smooth, shiny surface and a central crateriform ulceration with a keratin plug in the form of a horn-like projection. Skin papules were present in the right lateral canthal area and extended to the lateral border of the lower eyelid, measuring 1.3 cm. On palpation, the lesion was firm, movable, and tender. It was secondarily infected and had a history of rapid progression over the last 5 weeks.

The patient had a history of the same lesion at the vermillion border of the lower lip that was diagnosed as SCC at another medical center 6 years previously. The SCC was surgically treated by a V-wedge resection of the lower lip at that time. One and a half years later, the same lesion reappeared in the left lateral canthal area and extended over the lower eyelid. The patient did not seek medical treatment. He claimed that the psychological impact of the lip resection prevented him from seeking medical treatment. The patient stated that this lesion spontaneously disappeared after 5 months. One year later, a similar lesion reappeared on the right nasolabial fold and soon disappeared within a few weeks.

At the present presentation, the same lesion was observed in the right canthal area, extending to the lower eyelid. The patient was now seeking treatment because of pain secondary to infection of the lesion. The preliminary diagnosis of the lesion was a secondarily infected KA, and the differential diagnoses included SCC, verrucous carcinoma, and metastatic skin carcinoma.

**Figure 1:**
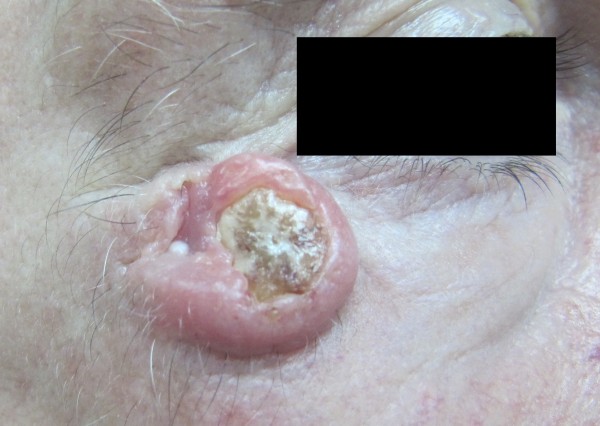
**Photograph of the lesion before surgical excision.** The lesion was a dome-shaped nodule with smooth, shiny surface and central crateriform ulceration with a keratin plug in the form of a horn-like projection. Skin papules were present in the right lateral canthal area (lower eyelid).

**Figure 2:**
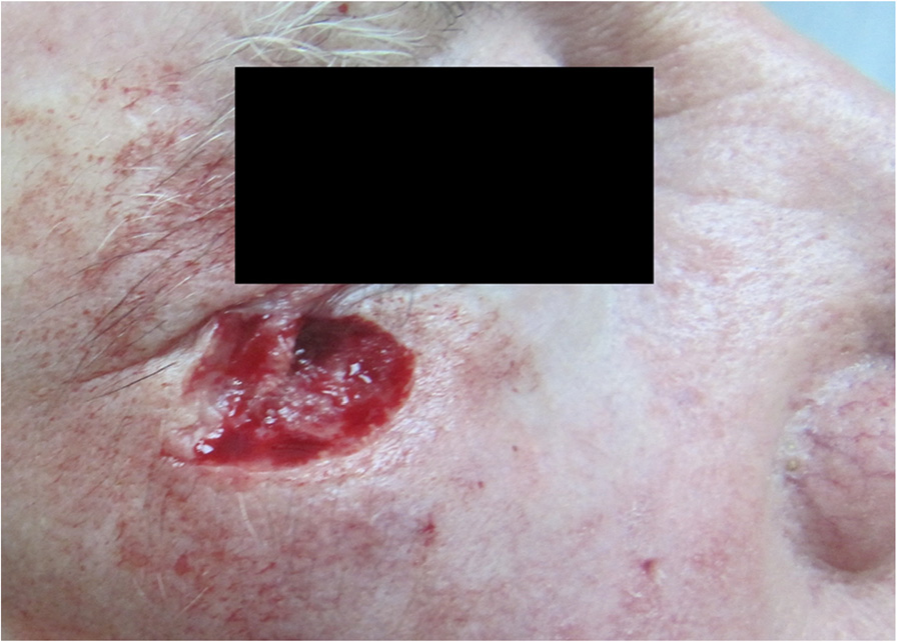
**Photograph of the wound after surgical excision.** A safe margin of 3 mm was established with preservation of the lateral canthal ligament and lower eyelid volume.

A clinical decision was made to approach the case surgically. Ectropion was considered as a possible postoperative complication. Excisional biopsy was performed under local anesthesia with 2% lidocaine and 1:100,000 epinephrine. The infraorbital and zygomaticofacial nerves were anesthetized. The patient was informed about all possible complications and provided written consent to undergo simple surgical excision and primary closure without the use of a local flap. A simple elliptical incision was created with a 3-mm safe margin, and local tissues were bilaterally advanced for closure [Figures 1 and 2]. The wound was closed primarily using Vicryl 4–0, and postoperative prophylactic antibiotic and analgesic medications were prescribed. The patient underwent suture removal 7 days later, and no complications were observed.

Histopathological examination of the biopsy specimen showed well-differentiated squamous epithelium with only a mild degree of pleomorphism from the basal layer to the surface keratin. However, dyskeratosis (abnormal or premature keratin production) was present as evidenced by individually keratinizing lesional cells and keratin pearls identical to those of well-differentiated SCC.

The correlation among the history, clinical picture, and histopathological findings strongly suggested KA. The patient was followed up for 6 months without recurrence.

## Conclusions

Ideal treatment of KA involves surgical resection with a safe margin of 3 to 5 mm. Medical treatment can be used for patients in whom surgical intervention is either not feasible or desirable for anatomical reasons or because of the patient’s systemic condition. Medical intervention may be appropriate in patients with multiple lesions, patients with lesions not amenable to surgery because of the lesion size or location, and patients with comorbidities that contraindicate the performance of a surgical procedure. The literature concerning medical intervention for KA is limited to case reports and of unproved. Systemic retinoids, such as isotretinoin, may be considered for patients with lesions too numerous for surgical intervention. Intralesional methotrexate [10], 5-fluorouracil, bleomycin, and steroids have been used with success [11]. Annest in 2002 reviewed the use of intralesional injection of methotrexate in 38 patients, 18 of whom showed a 92% rate of clinical resolution; however, the patients needed an average of two injections [10]. Dendorfer in 2003 used both topical imiquimod and 5-fluorouracil and achieved complete remission in 4 to 11 weeks [12]. KA is radiosensitive and responds well to low doses of radiation (< 10 Gy). Radiation therapy may be useful in select patients with large tumors in whom resection will result in a cosmetic deformity or in patients with tumors that have recurred following excisional surgery. Radiation therapy is less appealing in younger patients, in whom radiation damage worsens with time. Radiation therapy is an important alternative treatment for select patients who understand its risks and benefits and who are not good surgical candidates [13]. Both laser therapy and cryotherapy have been used successfully in patients with small KAs, in patients with KAs in locations difficult to treat surgically, and as an adjunct to surgical removal. The primary therapy for KA is currently surgical excision with adequate margins (3–5 mm) and histopathologic evaluation to exclude invasive SCC. A partial shave biopsy usually inadequately distinguishes KA from invasive SCC. Because the biological behavior of an individual KA cannot be predicted, many consider surgical treatment of KA to be equivalent to treatment for SCC. Mohs micrographic surgery may be indicated for large or recurrent KAs or for KAs located in anatomic areas with cosmetic or functional considerations.

The differentiation between KA and well-differentiated SCC may be problematic, particularly in cases of secondarily infected KAs, in which the histopathological picture may appear to be more aggressive in nature than is the actual KA. In such cases, the findings of the histopathological study, which is one of the most reliable diagnostic techniques, may direct the treatment of KA toward an unnecessary surgical procedure. Therefore, careful establishment of the correlation among the history, clinical findings, and histopathological picture is highly indicated in patients with KA, particularly in those with secondarily infected KAs, which may have lost the typical clinical characteristics of uninfected KAs. This is recommended to avoid unneeded major surgical intervention, possible surgical complications, and high costs.

## Consent

Written informed consent was obtained from the patient’s son (the patient was illiterate and unable to provide written consent, but provided verbal consent) for publication of this Case Report and any accompanying images. A copy of the written consent is available for review by the Editor-in-Chief of this journal.

## Competing interests

The author declares that he has no competing interests.
